# DeM-FCN: an ultra-lightweight and purely convolutional framework for edge-native human activity recognition in wearable fitness tracking

**DOI:** 10.3389/fspor.2026.1858408

**Published:** 2026-06-10

**Authors:** Yifeng Xu, Jiahao Li, Zhongwei Huang, Taiyu Cheng, Chao Chen, Baohua Tan, Zongming Tan, Hongbin Chen, Yuanye Zhou

**Affiliations:** 1 School of Science (School of Chip Industry), Hubei University of Technology, Wuhan, China; 2 National “111 Research Center” Microelectronics and Integrated Circuits, Hubei University of Technology, Wuhan, China; 3 Faculty of Engineering, University of Nottingham, Nottingham, United Kingdom; 4 School of Computer Science, Hubei University of Technology, Wuhan, China; 5 School of Mechanical and Electronic Engineering, Wuhan University of Technology, Wuhan, China; 6 School of Computer Science, University of Nottingham, Nottingham, United Kingdom; 7 Faculty of Science, University of Melbourne, Parkville, Vic, Australia; 8 Department of Respiratory and Critical Care Medicine, Renmin Hospital of Wuhan University, Wuhan, China; 9 School of Electrical and Electronic Engineering, Hubei University of Technology, Wuhan, China

**Keywords:** deep learning, edge computing, exercise biomechanics, human activity recognition, resistance training, wearable sensors

## Abstract

**Introduction:**

Wearable human activity recognition has become an important component of intelligent fitness tracking, but deploying accurate recognition models on resource-constrained edge devices remains challenging. Existing deep learning methods often rely on recurrent structures, attention mechanisms, or complex hybrid architectures, which increase computational cost and limit real-time deployment.

**Methods:**

This study proposes DeM-FCN, a lightweight and purely convolutional framework for smart dumbbell-based resistance-training activity recognition. The model integrates a physics-aware input representation, Gaussian noise regularization, stacked one-dimensional convolutional blocks, Global Max Pooling, and a cost-sensitive focal loss to improve subject-independent recognition. The input representation extends raw inertial measurements by introducing trigonometric encoding of Euler angles and acceleration and gyroscope magnitude features, allowing the model to capture both orientation-related motion patterns and orientation-insensitive motion intensity. The proposed model was evaluated using Leave-One-Subject-Out cross-validation on a custom smart dumbbell dataset containing four resistance-training exercises collected from 15 subjects.

**Results:**

DeM-FCN achieved an accuracy of 0.966, macro F1-score of 0.916, and macro AUC of 0.982, while maintaining only 73.7 K parameters, 14.84 M FLOPs, and a model size of 0.29 MB. Additional evaluations on PAMAP2 and MHEALTH suggested that the convolutional backbone retained useful class-ranking ability on public IMU-based HAR datasets, while the reduced macro F1-scores indicated that hard-label daily activity recognition remains more challenging than constrained resistance-training recognition due to broader activity diversity, sensor-domain differences, and missing modality information. A refined ablation study confirmed that trigonometric encoding and magnitude features provide complementary benefits, with magnitude features contributing more strongly to cross-subject robustness.

**Discussion:**

The results suggest that DeM-FCN provides a favorable accuracy-efficiency trade-off for wearable resistance-training recognition and offers a practical foundation for edge-oriented fitness monitoring.

## 1 Introduction

Worldwide trends in fitness and physical activity frequently evolve to influence market direction, as continuously observed by global surveys of health and fitness professionals (1). However, the rising popularity of physical recreation, such as aerobics and strength conditioning, brings an inherent risk of injury, underscoring an urgent need for epidemiological data and risk management strategies to ensure safe fitness environments (2). Concurrently, shifting global demographics—specifically increased life expectancy and declining birth rates—are resulting in an aging population that is increasingly prone to physical and cognitive declines (3). These dual challenges regarding fitness safety and pervasive healthcare monitoring have catalyzed the rapid development of continuous, technology-driven human behavior tracking systems.

To address these monitoring and training needs, early technological solutions focused on modernizing traditional physical education infrastructures. In university sports environments facing funding shortages and backward training methods, researchers successfully deployed agility training systems utilizing wireless *ad hoc* and ESP-MESH network topologies (4). These early systems demonstrated the capability of mesh networks to facilitate intra-group communication and transmit real-time data with near-zero packet loss and extremely low node response times (5). Experimental results indicated that college students employing these network-aided apparatuses exhibited significant improvements in rapid direction change, body coordination, and predictive decision-making (6). While effective for macroscopic agility tracking, these infrastructural systems lacked the localized granularity required for precise biomechanical and kinematic analysis.

To achieve fine-grained monitoring, the paradigm shifted toward wearable sensor-based Human Activity Recognition (HAR) coupled with advanced Deep Learning (DL) algorithms. Traditional HAR pipelines relied heavily on handcrafted features which proved inadequate for handling complex, high-dimensional sequences of motor movements (7). Early deep architectures overcame some of these bottlenecks by combining convolutional and LSTM recurrent units to model the temporal dynamics of multimodal sensor activations (8). Modern frameworks have further advanced this by utilizing deep architectures, such as CNN-ResBiGRU networks, to automatically extract unique spatio-temporal features from multimodal wearable sensors (e.g., electromyography and inertial measurement units), achieving exceptional classification accuracy across diverse gym workout activities (9). Furthermore, advancements in contrastive representation learning have demonstrated that incorporating supplementary modalities, such as bio-impedance sensing, during the training phase can significantly augment the inference capabilities of IMU-only fitness activity recognition models without requiring the secondary sensor during actual deployment (10). The development of standardized, multi-sensor public datasets capturing synchronized inertial and orientation data from various body locations (e.g., chest, hands, and knees) has further accelerated the objective evaluation of these robust machine learning pipelines (11).

Despite the high accuracy of deep neural networks, their extensive computational complexity poses significant challenges for deployment on resource-constrained wearable devices. Current cloud computing architectures hinder real-time responsiveness due to critical efficiency and latency issues; therefore, the convergence of edge computing and deep learning has emerged as a highly desirable solution (12). Unleashing artificial intelligence directly near the raw IoT sensor data sources is crucial for extracting accurate information in complex environments (13).

To realize this vision of “Edge DL” in HAR, extensive research has focused on model compression and the deployment of Tiny Machine Learning (TinyML). For example, modifying hybrid models by replacing computationally intensive BiLSTMs with residual LSTMs (ResLSTM) alongside data flipping augmentation has been shown to drastically reduce parameters while maintaining state-of-the-art context awareness (14). Similarly, explainable frameworks like X-LiteHAR have utilized adaptive EEMD for noise reduction and structured quantization to shrink model sizes by 70%, enabling sub-10 ms inference latencies on smartphones for clinical rehabilitation monitoring (15). In specific TinyML applications, researchers have deployed heavily quantized models, such as DeepConv LSTMs, onto resource-constrained microcontrollers (e.g., Arduino Nano 33 BLE Sense), achieving real-time HAR with memory footprints under 30 KB (16). TinyML has also enabled continuous cardiovascular exercise tracking (e.g., jumping jacks and squat jumps) directly from arm-worn IMUs, maintaining high precision while consuming less than 60 KB of memory (17). Other highly targeted edge applications include low-power embedded Systems-on-a-Chip attached to sports weights for off-the-person trajectory supervision (18), customized Raspberry Pi Pico boards running highly optimized XGBoost models for real-time psychological stress classification (19), and multi-modal edge systems merging IMUs and thermal cameras for end-to-end kitchen activity recognition at latencies near 25 ms (20).

Furthermore, deploying intelligent systems at the network edge inherently addresses the critical data privacy concerns associated with cloud reliance. Methodologies utilizing Federated Learning combined with Self-Organizing Maps (SOM) allow robust activity detection and the onboarding of new users without transmitting raw, sensitive HAR data (21). Recent IoT frameworks for sports safety monitoring have also successfully integrated CRNN spatiotemporal analysis with 8-bit model quantization and AES-128 secure transmission mechanisms, ensuring end-to-end data confidentiality while maintaining embedded inference latencies below 50 ms (22).

While the aforementioned methodologies have significantly advanced the field of wearable HAR and edge computing, a critical gap remains in developing an ultra-lightweight, highly accurate framework capable of isolating complex kinematics without relying on recurrent structures or heuristic windowing. Many current models still depend on recurrent units (like LSTMs) which, despite heavy compression, add computational overhead, or they utilize traditional static cycle segmentation techniques that are highly vulnerable to baseline drifts and varied sensor noise. To circumvent these inherent limitations, this paper proposes the DeM-FCN framework—an ultra-lightweight, purely convolutional architecture integrated with physics-aware kinematic feature engineering and an overlapping temporal sliding window mechanism utilizing Global Max Pooling. By eliminating recurrent layers and reducing reliance on heuristic action boundaries, DeM-FCN is designed to provide a favorable accuracy-efficiency trade-off for edge-oriented wearable resistance-training recognition.

## 2 Materials and methods

### 2.1 Model motivation and overall architecture overview

**Figure 1:**
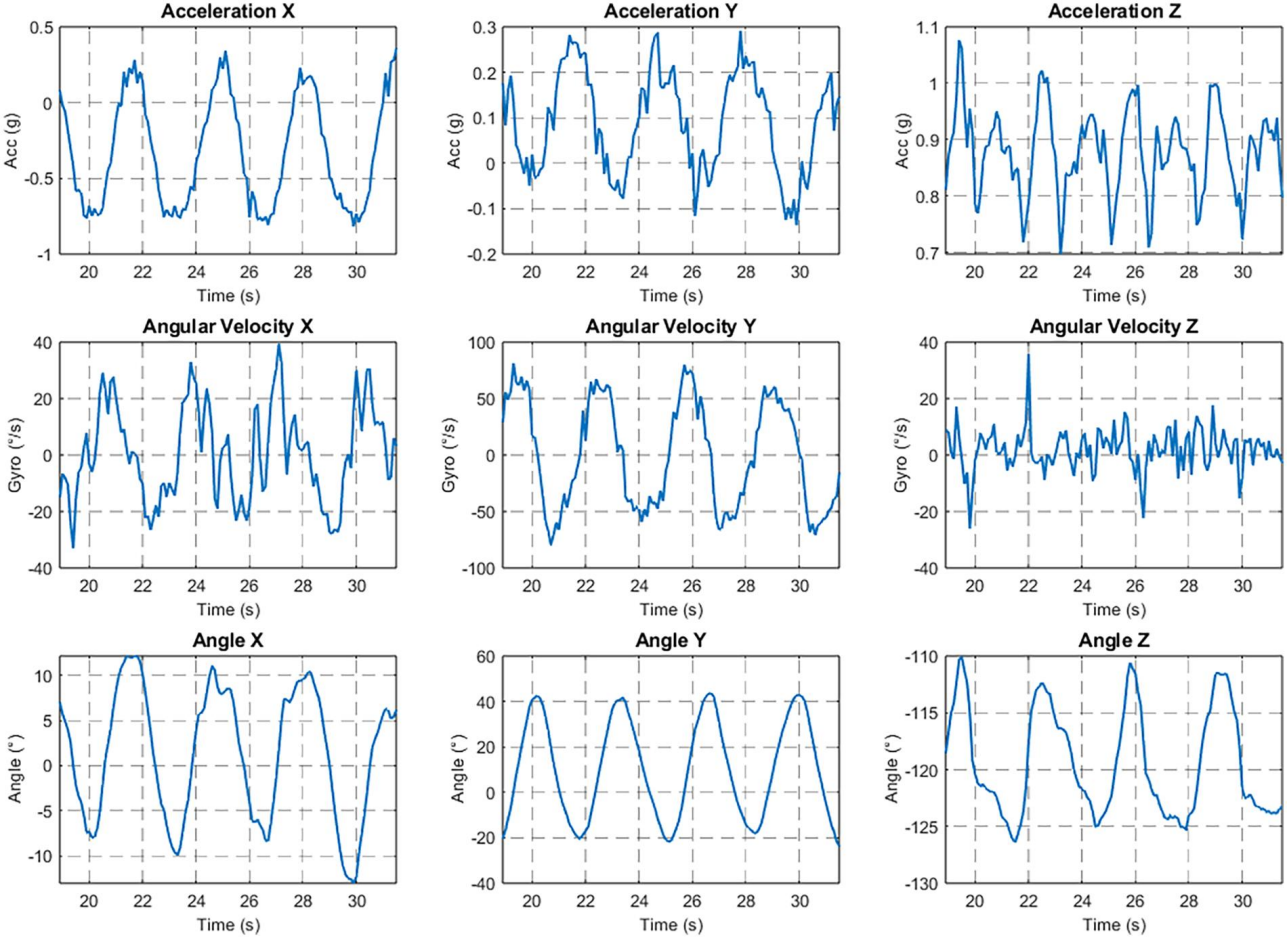
The 9-axis raw sensor signals acquired during the lateral raise exercise reveal the kinematic 248 distinctions among different axes.

This study involved four different upper-body strength training exercises using dumbbells: Bicep Curl (BC), Lateral Raise (LR), Shoulder Press (SP), and Tricep Extension (TE). To visually demonstrate the spatial complexity of the movements, Figure 1 presents a representative segment of the 9-axis raw sensor signals recorded during a lateral raise. As observed, while the periodic nature of the motion is evident, the signal amplitude, baseline drift, and noise distribution vary drastically across different sensor modalities and spatial axes. Furthermore, because the resting baseline of angular velocity is intrinsically zero—whereas the baselines for acceleration and Euler angles are non-zero due to gravity and spatial orientation—traditional static cycle segmentation methods relying on a single, predefined axis are highly prone to failure.

**Figure 2:**
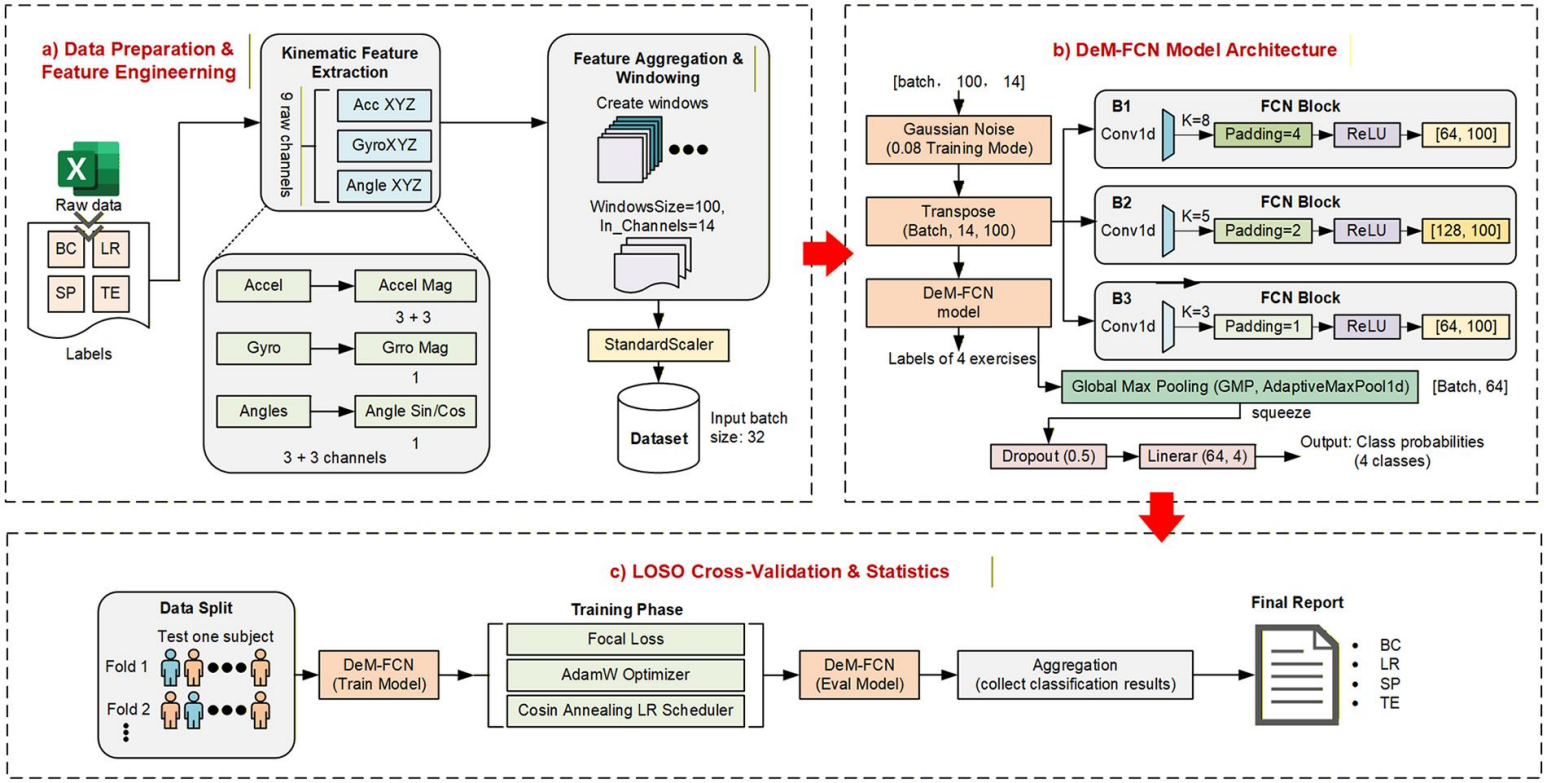
Overview of the proposed Dem-FCN–based experimental framework.

To circumvent the inherent limitations of traditional heuristic-based cycle segmentation, our proposed DeM-FCN framework (Figure 2) shifts the paradigm from explicit temporal cropping to continuous, data-driven feature extraction. a) Data preparation and feature engineering raw sensor data are transformed into kinematic features (acceleration, gyroscope, and angle information), followed by feature aggregation using sliding windows and standardization. b) DeM-FCN model architecture the input sequences are processed through multiple 1D convolutional blocks with different kernel sizes, followed by global max pooling, dropout, and a fully connected layer to produce class probabilities. c) LOSO (Leave-One-Subject-Out) cross-validation and evaluation the dataset is split subject-wise, the model is trained using focal loss and AdamW optimizer with cosine annealing, and final performance is obtained by aggregating classification results across all folds.

Firstly, to address the heterogeneous baselines and amplitude variations across different modalities, we implement a robust preprocessing and feature engineering pipeline. Rather than relying on a single raw axis, we compute the Euclidean magnitudes of acceleration and angular velocity, which provide orientation-invariant metrics of movement intensity. Furthermore, the continuous stream is segmented using a sliding window approach with a fixed length and overlap, eliminating the dependency on predefined zero-crossing points. Global standardization (StandardScaler) is subsequently applied to enforce a zero-mean and unit-variance distribution across all channels, effectively neutralizing the intrinsic baseline discrepancies between the gyroscope and other sensors.

Secondly, the architectural design of the DeM-FCN inherently resolves spatial complexity and noise distribution issues. A Gaussian noise injection layer is introduced at the input stage to simulate sensor perturbations, functioning as an explicit regularizer that enhances the model's tolerance to dynamic baseline drifts. The core feature extraction is driven by stacked Fully Convolutional Network (FCN) blocks with decreasing kernel sizes (13, 17, 18). These 1D convolutional layers treat the 14-channel input as a unified multivariate spatial representation, automatically capturing the intricate cross-channel correlations without requiring manual axis alignment. The internal Batch Normalization layers further stabilize the internal covariate shifts caused by multi-sensor fusion. Finally, a Global Max Pooling (GMP) layer replaces traditional flattening operations. GMP acts as a translation-tolerant temporal aggregator. When salient kinematic features occur at different temporal positions within a sliding window, GMP can help preserve the strongest local responses, thereby reducing the model's dependence on exact cycle-boundary alignment.

### 2.2 Data acquisition

The physical layer is engineered to ensure high-fidelity kinematic capture while eliminating the spatial restrictions of wired connections.

#### 2.2.1 Dataset description and kinematic modalities

The experimental data utilized in this study comprises multivariate time-series kinematic signals acquired via wearable Inertial Measurement Units (IMUs). For each continuous motion, the sensors recorded 9-axis temporal sequences at a constant sampling rate, encapsulating triaxial linear acceleration (measured in *g*), triaxial angular velocity (measured in°/s), and triaxial spatial orientation (Euler angles in degrees). Data was collected from a diverse cohort of 15 subjects to ensure a robust representation of inter-subject biomechanical variances.

#### 2.2.2 Hardware implementation

**Figure 3:**
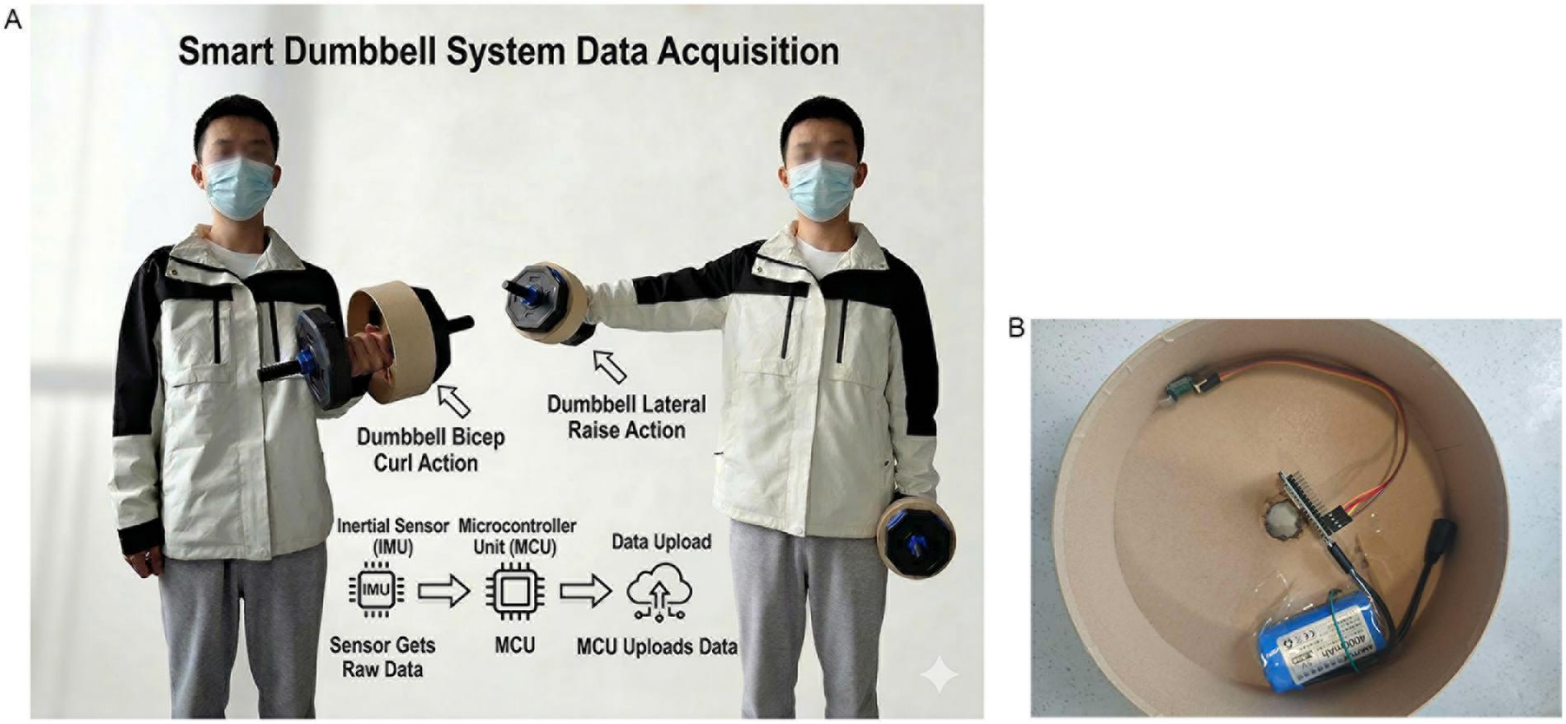
**(A)** Data acquisition setup of the smart dumbbell system. **(B)** Data acquisition setup of the smart dumbbell system.

The sensing node utilizes the JY901S 9-axis inertial measurement unit. Built upon the MPU6050 core architecture, it integrates a 3-axis accelerometer, a 3-axis gyroscope, and a 3-axis magnetometer, providing calibrated high-precision attitude data. For wireless communication, we employ the ESP8266-NodeMCU Wi-Fi module. To ensure stable throughput, the module is configured in STA Mode, connecting to a local Wi-Fi router to form a Star Topology network. This setup allows for the transparent transmission of sensor payloads to the designated port of the receiving terminal. Figure 3 shows the data collection and the collection module. (a) demonstration of the exercise scenarios (Bicep Curl and Lateral Raise) and the wireless transmission workflow; (b) internal hardware components of the IoT sensor node, integrating a 9-axis IMU (JY901S), a Wi-Fi microcontroller (ESP8266), and a lithium-ion battery.

### 2.3 Data preparation and feature engineering

Let $S\in R^{T\times 9}$ denote the raw physical signal sequence collected by the Inertial Measurement Unit (IMU) within a temporal window of length *T*. At any given time step *t*, the raw feature vector can be represented as:

$$\begin{array}{c} s^{\left(t\right)}={\left[a_{x}^{\left(t\right)},\;a_{y}^{\left(t\right)},\;a_{z}^{\left(t\right)},\;\omega_{x}^{\left(t\right)},\;\omega_{y}^{\left(t\right)},\;\omega_{z}^{\left(t\right)},\;\theta_{x}^{\left(t\right)},\;\theta_{y}^{\left(t\right)},\;\theta_{z}^{\left(t\right)}\right]}^{⊤} \end{array}$$

where $a^{\left(t\right)}\in R^{3}$ represents the 3-axis acceleration, $\omega^{\left(t\right)}\in R^{3}$ denotes the 3-axis angular velocity, and $\theta^{\left(t\right)}\in R^{3}$ indicates the 3-axis Euler angles.

Directly feeding raw Euler angles into a neural network introduces a severe mathematical vulnerability: Gimbal Lock and Boundary Discontinuity. In 3D spatial rotation, the domain of Euler angles is typically bounded within [−π,π]. When a user performs an exercise that crosses the vertical anatomical plane of the body, such as a Shoulder Press, the sensor angle frequently undergoes an instantaneous, non-continuous inversion from $+180^{\circ }$ directly to $-180^{\circ }$. This mathematical discontinuity forces the network to generate artificially massive, spurious gradients ∇θ during temporal differencing or local convolution, thereby completely destroying the topological manifold of the feature space.

To eliminate this non-continuity, we introduce an Anatomical Mapping mechanism in the preprocessing layer. Using trigonometric functions, the Euler angles are projected from a discontinuous scalar space into a continuous, two-dimensional orthogonal circular space:

$$\begin{array}{c} {\tilde{\theta }}_{d}^{\left(t\right)}={\left[\sin\left(\frac{\pi }{180}\theta_{d}^{\left(t\right)}\right),\;\cos\left(\frac{\pi }{180}\theta_{d}^{\left(t\right)}\right)\right]}^{⊤},\;\forall d\in x,y,z \end{array}$$

The reconstructed six-dimensional continuous angle feature ${\tilde{\Theta }}^{\left(t\right)}\in R^{6}$ preserves the Absolute Spatial Reference Frame relative to the human torso, granting the network unambiguous spatial awareness of the arm's current gravity projection distribution.

Furthermore, to eradicate the base-coordinate drift interference caused by different subjects’ gripping rotation habits, we extract the Euclidean ($L_{2}$) norm of both the acceleration and angular velocity. This serves as an Orientation-Invariant kinematic scalar feature:

$$\begin{array}{c} m_{a}^{\left(t\right)}=\;\|a^{\left(t\right)}{\|}_{2}\;=\;\sqrt{{\left(a_{x}^{\left(t\right)}\right)}^{2}+{\left(a_{y}^{\left(t\right)}\right)}^{2}+{\left(a_{z}^{\left(t\right)}\right)}^{2}}\; \end{array}$$

$$\begin{array}{c} m_{\omega }^{\left(t\right)}\;=\;\|\omega^{\left(t\right)}{\|}_{2}\;=\;\sqrt{{\left(\omega_{x}^{\left(t\right)}\right)}^{2}+{\left(\omega_{y}^{\left(t\right)}\right)}^{2}+{\left(\omega_{z}^{\left(t\right)}\right)}^{2}} \end{array}$$

Ultimately, through feature concatenation, the variables at each time step are decoupled and reconstructed into a 14-dimensional purified kinematic feature vector $x^{\left(t\right)}\;=\;[a^{\left(t\right)};\omega^{\left(t\right)};{\tilde{\Theta }}^{\left(t\right)};m_{a}^{\left(t\right)};m_{\omega }^{\left(t\right)}{]}^{⊤}\in R^{14}$. By applying a sliding window segmentation, the final input tensor for the model is formalized as $X\in R^{W\times 14}$, where *W* represents the number of frames within the temporal window. Consequently, the original 9-dimensional raw channels were expanded into a 14-dimensional kinematic feature space. To format the continuous data stream for the DeM-FCN architecture, an overlapping sliding-window segmentation protocol was employed, with a window size of 100 frames and a step size of 50 frames, yielding 50% overlap. This setting was selected as a practical compromise between temporal coverage, boundary robustness, sample efficiency, and inference frequency. A 100-frame window provides sufficient temporal context to cover the main motion phase of a resistance-training repetition, while avoiding an overly long window that may increase response delay and dilute short discriminative peaks. The 50-frame step reduces sensitivity to action-boundary uncertainty and increases the number of training windows under the limited subject cohort. This procedure constructs a localized spatio-temporal tensor of 14 × 100 for each segmented action instance, which is subsequently normalized to zero mean and unit variance using global standardization to reduce amplitude disparities across sensor modalities. Although this window configuration was kept fixed across all experiments for fair comparison, systematic sensitivity analysis of window size and overlap ratio was not conducted in the present study.

### 2.4 Decoupled motion fully convolutional backbone

Considering the stringent computational constraints of wearable edge devices and the high risk of overfitting in small-sample scenarios, DeM-FCN Abandons Recurrent Neural Networks (RNN/LSTM) laden with complex gating units. Recurrent networks (RNNs/LSTMs) suffer from inherent sequential dependence: each time-step output relies on the previous hidden state, which prevents parallel computation and results in O(T) inference complexity. By contrast, 1D-CNNs process all time steps in parallel, enabling vectorized hardware acceleration with O (19) complexity. This bottleneck is further amplified on low-frequency edge microcontrollers. Moreover, RNNs require persistent hidden-state storage, increasing memory bandwidth and power consumption during continuous inference. Instead, we constructed a highly efficient temporal backbone based on a Fully Convolutional Network (FCN).

At the input terminal of the network, to enhance the model's robustness against cross-subject domain shifts, we designed a Gaussian Noise Injection mechanism. During the forward propagation in the training phase, a layer of microscopic random noise is superimposed onto the input tensor:

$$\begin{array}{c} \tilde{X}=X+Z,Z∼N(0,\sigma^{2}I) \end{array}$$

where the standard deviation is empirically set to σ=0.08. Physically, this operation simulates the Physiological Muscle Tremors of diverse users as well as the inherent thermal noise of the sensors. Functioning as a potent stochastic regularizer, it coerces the network into learning the macroscopic envelope of the motion trajectory, rather than memorizing the microscopic, subject-specific fluctuations of the training set.

The feature extraction backbone is built by stacking three 1D Convolutional Blocks (FCN Blocks) with progressively altering Receptive Fields. For the *l*-th convolutional block, given the input feature map $H^{\left(l-1\right)}$, its feature mapping process is defined as:

$$\begin{array}{c} H^{\left(l\right)}=\delta (\mathrm{BN}(W^{\left(l\right)}*H^{\left(l-1\right)}+b^{\left(l\right)})) \end{array}$$

where ∗ denotes the 1D causal convolution operation, and $W^{\left(l\right)}$ and $b^{\left(l\right)}$ are the convolutional kernel weights and biases for the respective layer. The kernel sizes across the three layers are set to k∈{8,5,3}. BN(⋅) represents Batch Normalization, utilized to eliminate internal covariate shifts, and δ(⋅) is the ReLU activation function, defined as δ(x)=max(0,x).

In the multi-scale feature aggregation phase, the academic consensus typically relies on Global Average Pooling (GAP) to flatten the temporal dimension. However, the mathematical expectation of GAP is uniformly distributed across the entire temporal window W:

$$\begin{array}{c} v_{GAP}=\frac{1}{W}\overset{W}{\underset{t=1}{\sum }}\;\;H_{*,t}^{\left(L\right)} \end{array}$$

In fitness activity recognition, motion sequences frequently contain prolonged periods of pre-motion rigidity or static pauses at the peak of contraction. These resting frames severely dilute the activation values of the genuine exertion moments. To counter this, DeM-FCN innovatively deploys Global Max Pooling (GMP) as a superior alternative to GAP:

$$\begin{array}{c} v_{GMP}=\underset{1\leq t\leq W}{\max}\;H_{*,t}^{\left(L\right)} \end{array}$$

Mathematically, GMP functions as a hard-attention selection mechanism. It entirely disregards redundant pause frames with low activation, precisely capturing the Transient Peak Features that possess the highest kinetic energy within the 2-second window. This mechanism drastically magnifies the local topological discrepancies between highly similar exercises (e.g., Shoulder Press versus Bicep Curl) at their points of maximum exertion.

### 2.5 Cost-sensitive focal loss with difficulty-aware class weighting

The four resistance-training exercises differ not only in sample distribution but also in biomechanical classification difficulty. Under the subject-independent LOSO setting, some classes are more difficult to distinguish because of larger inter-subject execution variability and greater motion-pattern overlap. In particular, Shoulder Press and Lateral Raise involve shoulder-dominant movements with larger freedom in movement amplitude, elbow flexion, torso compensation, and lifting trajectory. By contrast, Bicep Curl and Tricep Extension are relatively more constrained elbow-dominant movements and usually produce more stable kinematic patterns.

To account for this difficulty imbalance, we used a cost-sensitive focal loss. The focal term reduces the contribution of easy and high-confidence samples, while increasing the optimization focus on difficult or low-confidence samples. A class-specific weighting term was further introduced to emphasize classes with higher biomechanical variability and greater confusion risk. The loss function is formulated as:

$$L_{\mathrm{CS}\text{-}\mathrm{FL}}=-\frac{1}{N}\overset{N}{\underset{i=1}{\sum }}\;w_{y_{i}}(1-p_{i,y_{i}}{)}^{\gamma }\log(\;p_{i,y_{i}})$$

where *N* is the batch size, $y_{i}\in \{0,1,2,3\}$ is the ground-truth class label, and γ is the focusing parameter (set to 2.0).

In this study, the class-weight vector was empirically set to:

α=[0.8,1.0,4.0,1.2]

corresponding to BC, LR, SP, and TE, respectively. This setting was not intended as a simple correction for sample-count imbalance. Instead, it was designed as a difficulty-aware weighting strategy based on the biomechanical complexity and confusion risk of each exercise. A higher weight was assigned to Shoulder Press because it involves overhead movement, larger shoulder-joint freedom, and stronger inter-subject execution variability. A lower weight was assigned to Bicep Curl because its elbow-flexion pattern is relatively constrained and more stable across subjects. The remaining classes were assigned intermediate weights according to their expected movement variability.

It should be noted that this class-weight vector is an empirical configuration for the present smart dumbbell dataset rather than a universally optimal setting. The purpose of the weighting strategy is to guide the model to pay more attention to difficult classes during training. The effect of this loss configuration was further examined in the ablation study by replacing it with standard cross-entropy loss.

### 2.6 Experiments and evaluation strategy

In wearable human activity recognition, data generated by the same subject exhibits severe biometric autocorrelation. Employing a standard randomized train-test split (e.g., an 80/20 split) inadvertently allows the model to “memorize” a user's specific physiological traits (e.g., arm length, baseline strength) present in both sets, leading to data leakage and artificially inflated accuracy. To objectively evaluate the model's true generalization capability on edge devices, this study adopted the rigorous Leave-One-Subject-Out Cross-Validation (LOSO-CV) protocol. In each of the 15 experimental folds, all data from a single subject was strictly isolated to serve as the “unseen” test set, while the data from the remaining 14 subjects constituted the training set. This protocol simulates the real-world deployment scenario where a pre-trained wearable device is utilized by a completely novel, unregistered user. To strictly prevent feature distribution leakage, the standard scaler (StandardScaler) was fitted exclusively on the training set of the current fold. The derived mean and variance parameters were subsequently applied to transform the isolated test set. The final evaluation metrics reported are the Macro Average and Standard Deviation across all 15 folds.

The network was trained using the AdamW optimizer, which decouples weight decay from the gradient updates to provide superior L2 regularization, preventing overfitting on the small-scale dataset. The base learning rate was set to 0.002, weight decay to 0.01, and batch size to 32. The model was trained for 80 epochs per fold.

To optimize the convergence trajectory, we implemented a Cosine Annealing Learning Rate (Cosine Annealing LR) scheduler. The learning rate decays smoothly following a cosine curve from the initial maximum value down to a minimum threshold ($\eta_{min}=1\times 10^{-5}$). During the early stages of training, the relatively large learning rate acts as a kinetic momentum, assisting the model in escaping suboptimal local minima within the highly non-convex loss landscape. In the final epochs, the oscillatory decay to near-zero learning rates allows the network to perform ultra-fine gradient descent, precisely settling at the global optimum. This dynamic scheduling significantly bolstered the model's capacity to navigate the complex topological space of inter-subject variability. Table 1 summarizes the core hyperparameters of DeM-FCN, including model architecture and training settings.

**Table 1:** The core hyperparameters of DeM-FCN.

Category	Hyperparameter	Value
Model architecture	Number of convolutional blocks	3
	Kernel sizes	8, 5, 3
	Pooling	Global Max Pooling (GMP)
	Activation	ReLU
	Batch Normalization (BN)	Yes
	Gaussian noise std (*σ*)	0.08
Training	Optimizer	AdamW
	Initial learning rate	0.002
	Weight decay	0.01
	Batch size	32
	Epochs per fold	80
	LR scheduler	Cosine Annealing (min LR 1 × 10⁻⁵)
	Loss function	Cost-sensitive Focal Loss (*γ* = 2.0, class weights [0.8,1.0,4.0,1.2)

## 3 Results and analysis

### 3.1 Experimental results and analysis: subject-independent generalization and kinematic evaluation

#### 3.1.1 Overall generalization performance

To rigorously evaluate the generalization capability of the proposed DeM-FCN model on unseen users, we employed the Leave-One-Subject-Out (LOSO) cross-validation strategy. In this paradigm, the model is iteratively trained on N−1 subjects and tested on the single remaining subject. This approach prevents data leakage and authentically simulates real-world deployment where the system must analyze kinematics from novel users with unique biomechanical profiles.

As presented in Table 2, the DeM-FCN model demonstrates remarkable robustness. Out of the 15 subjects evaluated, the model achieved flawless predictions (Overall Accuracy = 1.0, Macro F1 = 1.0) for more than half of the cohort (8 subjects: GQ, LC, CX, HYJ, LY, WJH2, YHX, YW). Furthermore, the overall accuracy across all folds remained high, demonstrating that the fully convolutional architecture effectively captures translation-invariant kinematic features regardless of individual anatomical differences.

**Table 2:** Detailed subject-level performance in leave-one-subject-out (LOSO) cross.

Fold	Test subject	Train samples	Test samples	Overall accuracy	Macro precision	Macro recall	Macro F1	Macro AUC
1	GQ	180	30	1	1	1	1	1
2	LC	168	42	1	1	1	1	1
3	LJH	157	53	0.887	0.912	0.906	0.889	1
4	QZ	191	19	0.842	0.893	0.812	0.782	0.85
5	XYF	169	41	0.976	0.975	0.975	0.974	0.999
6	YWH	185	25	0.72	0.615	0.75	0.658	1
7	CX	154	26	1	1	1	1	1
8	HYJ	166	14	1	1	1	1	1
9	LY	157	23	1	1	1	1	1
10	TP	166	14	0.857	0.917	0.833	0.825	1
11	WJH	157	23	0.957	0.958	0.958	0.955	1
12	WJH2	168	12	1	1	1	1	1
13	YHX	157	23	1	1	1	1	1
14	YW	157	23	1	1	1	1	1
15	ZH	158	22	0.727	0.636	0.714	0.657	0.885

However, the LOSO evaluation also reveals the inherent challenges of subject-independent human activity recognition. The model encountered performance degradations for specific individuals, most notably YWH (Overall Accuracy: 0.720, Macro F1: 0.658) and ZH (Overall Accuracy: 0.727, Macro F1: 0.657). This variance is an expected phenomenon in biomechanical data analysis. Individual differences in limb length, muscle activation patterns, and exercise execution styles—such as variations in the range of motion or compensatory movements due to fatigue—introduce significant covariate shifts in the test distribution. Despite these drops in hard classification metrics, the macro AUC remained high for several low-performing subjects, including YWH and TP. This indicates that AUC and hard-label metrics capture different aspects of model behavior. Therefore, the discrepancy between macro AUC and macro F1-score is analyzed cautiously in the following heatmap-based discussion, with emphasis on ranking behavior rather than definitive probability calibration.

#### 3.1.2 Action-specific kinematic complexity analysis

Table 3 provides a granular breakdown of the model's performance across the four distinct exercises. The aggregated metrics (Mean ± Std) elucidate the varying degrees of kinematic complexity and inter-subject execution variance inherent to each movement.

**Table 3:** Action-level performance metrics aggregated across all subjects (mean ± standard deviation).

Action	Accuracy	Precision	Recall	F1	AUC
BC	0.969 ± 0.079	0.942 ± 0.158	0.990 ± 0.037	0.957 ± 0.105	0.997 ± 0.012
LR	0.950 ± 0.083	0.927 ± 0.258	0.803 ± 0.337	0.835 ± 0.301	0.939 ± 0.161
SP	0.959 ± 0.082	0.870 ± 0.278	0.927 ± 0.258	0.891 ± 0.261	0.993 ± 0.026
TE	0.985 ± 0.059	0.970 ± 0.117	1.000 ± 0.000	0.980 ± 0.076	1.000 ± 0.000

The Tricep Extension (TE) achieved the highest and most stable performance, with an extraordinary Recall of 1.000±0.000 and an AUC of 1.000±0.000. From a biomechanical perspective, the tricep extension is primarily a single-joint, one-dimensional motion restricted to the elbow hinge. The degrees of freedom are strictly limited, resulting in highly consistent spatial-temporal sensor signals across different subjects. Consequently, the DeM-FCN model easily isolates the deterministic feature representations for TE.

Similarly, the Bicep Curl (BC) exhibits excellent performance (Recall: 0.990±0.037, F1: 0.957±0.105). Like TE, BC is largely an elbow-hinge movement, which explains its high recall. The slightly lower precision (0.942) suggests that the model occasionally misclassifies other exercises as BC. This is likely due to the “spillover” of arm flexion features from complex exercises when subjects perform them with improper form.

Conversely, the Lateral Raise (LR) and Shoulder Press (SP) present significant challenges, evidenced by higher standard deviations in their metrics. LR exhibits the lowest average Recall (0.803±0.337) and F1-Score (0.835±0.301). The lateral raise involves the highly mobile glenohumeral joint (shoulder complex). Subjects frequently utilize compensatory mechanisms during LR, such as utilizing torso momentum, altering elbow flexion angles, or heavily engaging the upper trapezius. These subjective execution styles introduce immense noise into the kinematic data, making it difficult for the model to define a universal decision boundary. Similarly, SP shows the lowest Precision (0.870±0.278). Overhead pressing requires complex scapulohumeral rhythm; variations in shoulder mobility among subjects lead to drastically different spatial trajectories, confusing the model's spatial feature extractors.

#### 3.1.3 Confusion pattern and inter-subject variability analysis

**Figure 4:**
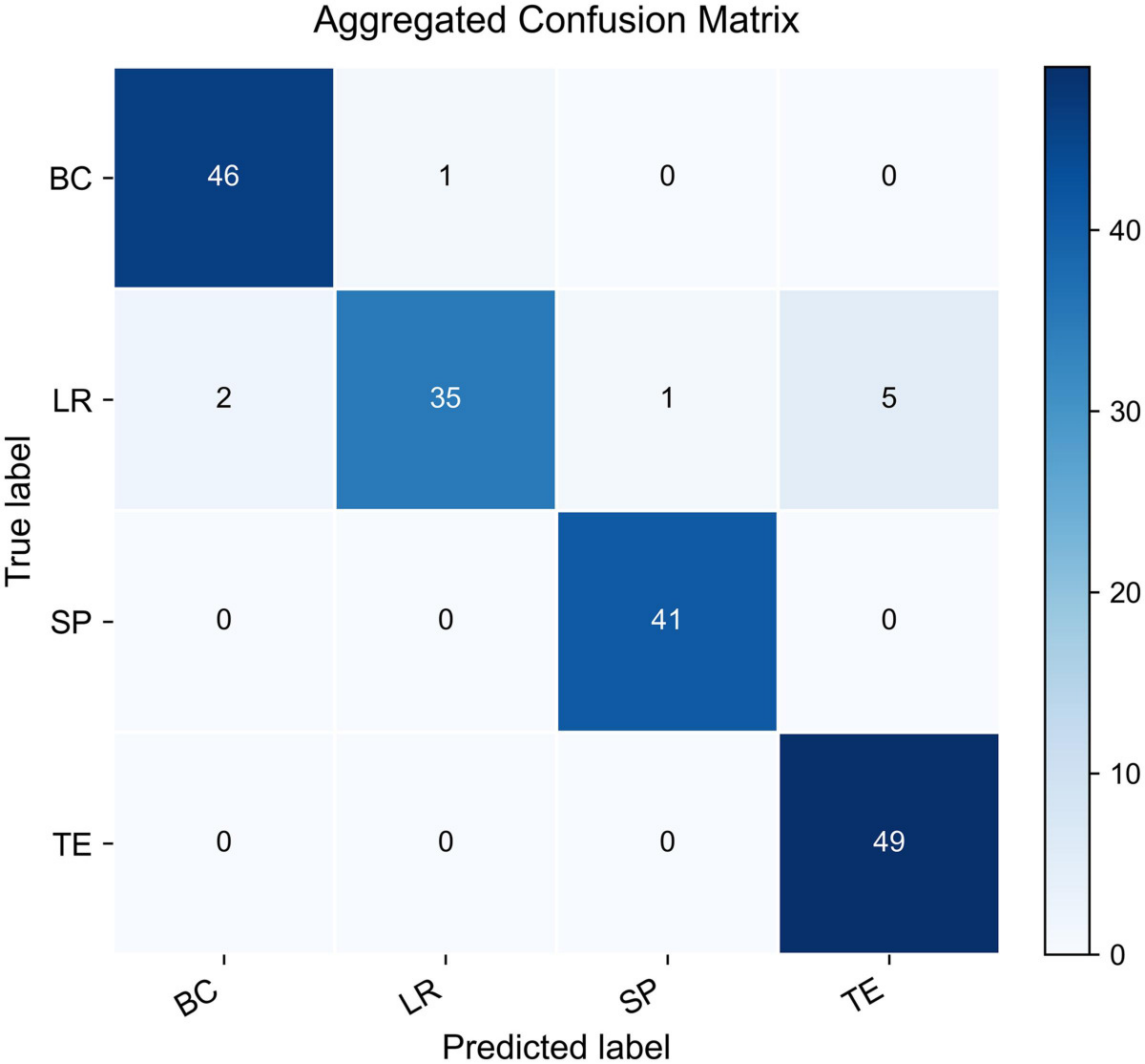
Aggregated confusion matrix across all leave-one-subject-out folds on the custom smart dumbbell dataset.

To further examine whether the perfect scores observed in several subjects were caused by task simplicity or potential overfitting, an aggregated confusion matrix was calculated across all Leave-One-Subject-Out folds, as shown in Figure 4. The matrix provides a global view of prediction patterns across all held-out subjects and helps identify the main sources of classification errors.

The results show that the errors were not randomly distributed across classes. Bicep Curl was recognized with a normalized correct prediction rate of 0.98, with only 0.02 of its samples misclassified as Lateral Raise. Shoulder Press and Tricep Extension were both recognized in the aggregated matrix, indicating that these two activities formed highly separable kinematic patterns in the current smart dumbbell setting. In contrast, Lateral Raise was the most challenging activity, with a correct prediction rate of 0.81. Its misclassified samples were mainly assigned to Tricep Extension, Bicep Curl, and Shoulder Press, with proportions of 0.12, 0.05, and 0.02, respectively.

This confusion pattern suggests that the main difficulty of the task lies in shoulder-related and transition-sensitive movement patterns rather than in random prediction errors. Lateral Raise is more susceptible to individual execution differences, such as variations in elbow flexion, shoulder abduction amplitude, torso compensation, and the use of momentum during lifting. These factors may shift its inertial pattern toward other upper-limb movements in some unseen subjects. By contrast, Shoulder Press and Tricep Extension exhibited more stable separability in the aggregated prediction space, which may explain the perfect scores observed for several subjects.

#### 3.1.4 Cautious interpretation of Auc-F1 discrepancy in misclassified samples

**Figure 5:**
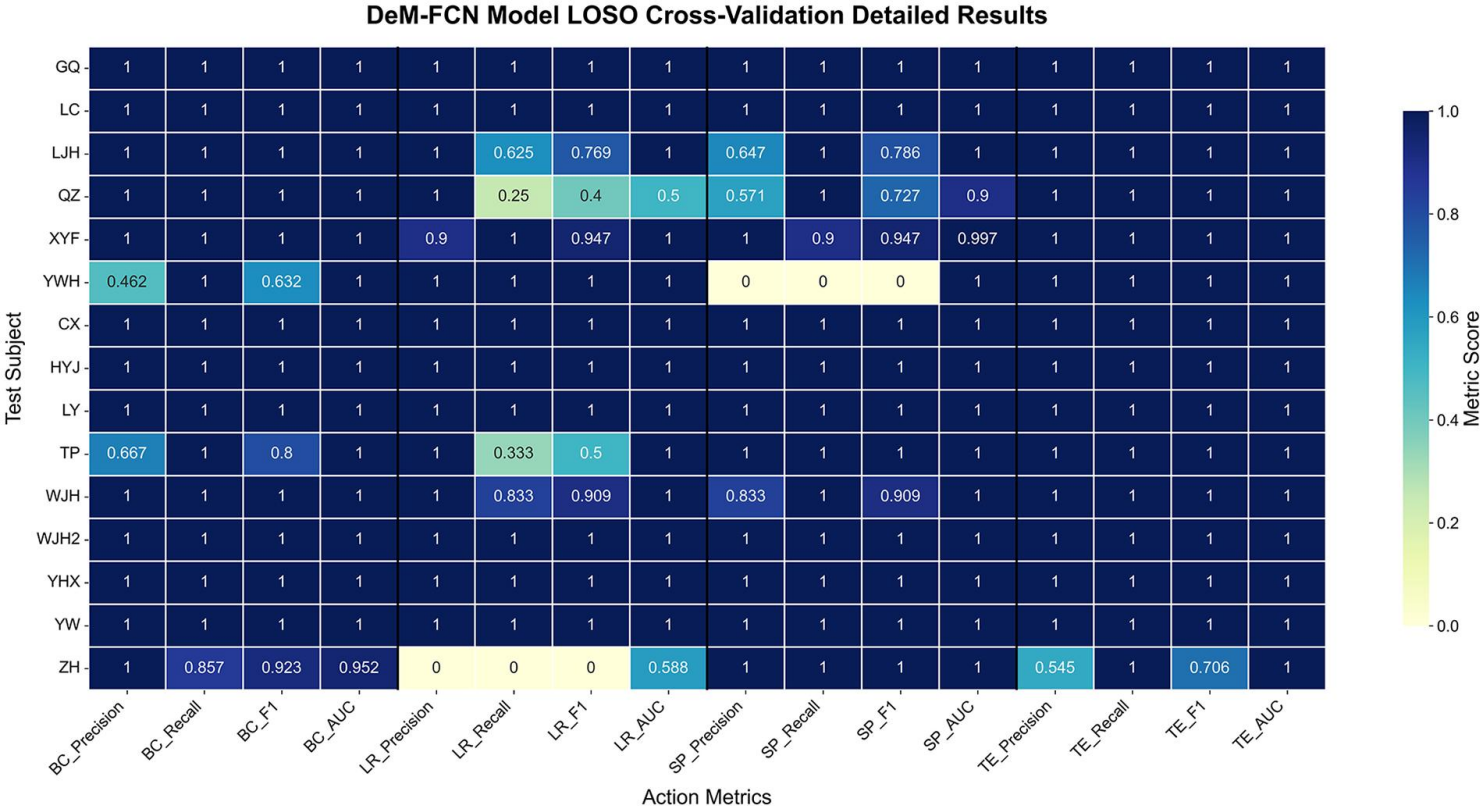
Heatmap of detailed classification metrics (precision, recall, F1-score, AUC) for each subject and action.

Figure 5 presents the fold-level heatmap of class-wise metrics and highlights several cases where AUC and hard-label metrics diverge. For subject YWH, the Precision, Recall, and F1-score for Shoulder Press were all 0, whereas the corresponding AUC reached 1.0. A similar but less extreme pattern was observed for subject ZH in Lateral Raise, where the F1-score was 0 and the AUC was 0.588. These cases suggest that soft-score ranking and final hard-label prediction may behave differently for some unseen subjects.

This discrepancy can be partly understood from the difference between hard-label metrics and ranking-based metrics. Precision, Recall, Accuracy, and F1-score are calculated from final class labels. In a multiclass classifier, the predicted label is usually determined by the argmax operation over the output probabilities. For example, if an unseen subject performs Shoulder Press with atypical kinematics, the model may assign a probability of 0.35 to Shoulder Press and 0.45 to Bicep Curl. In this illustrative case, the final prediction would be Bicep Curl rather than Shoulder Press, leading to zero true positives for Shoulder Press and consequently zero Precision, Recall, and F1-score for that class.

In contrast, AUC is computed from continuous class scores and evaluates the ranking of positive samples against negative samples under a one-vs-rest setting. Therefore, a high AUC for Shoulder Press in subject YWH suggests that the model assigned relatively higher Shoulder Press scores to true Shoulder Press samples than to non-Shoulder Press samples. However, this ranking advantage does not necessarily guarantee that Shoulder Press obtains the highest probability among all classes after the argmax operation.

This observation suggests that DeM-FCN may preserve useful class-ranking information even when the final hard-label decision is incorrect. However, this should not be interpreted as direct evidence that the learned feature space is fully separable or that the predicted probabilities are well calibrated. The discrepancy may be related to several factors, including class overlap, subject-specific distribution shifts, threshold sensitivity, and potential probability miscalibration. Since explicit calibration analysis was not performed in this study, we interpret this phenomenon cautiously as a difference between ranking-based evaluation and hard-label classification rather than as a confirmed calibration mechanism.

This observation points to a potential direction for future deployment. Lightweight personalization or calibration strategies, such as threshold adjustment, Platt scaling, temperature scaling, or few-shot subject adaptation, may help reduce the gap between ranking-based scores and hard-label predictions. However, their effectiveness needs to be empirically evaluated in future work.

### 3.2 Ablation study

#### 3.2.1 Disentangled validation of physics-aware feature engineering

To provide a more rigorous validation of the proposed feature engineering strategy, we designed the ablation study by separately evaluating trigonometric encoding and magnitude-based features. The experimental configurations and quantitative results are reported in Tables 4, 5, respectively. For all feature-level ablations, the input dimensionality was fixed to 14 channels, and removed feature components were replaced by zero-padding. Therefore, the convolutional backbone, parameter count, and training protocol remained identical across feature variants. This controlled setting allows the performance differences to be attributed mainly to the removed feature components rather than to changes in model capacity.

**Table 4:** Experimental configurations of the ablation variants.

Variant	Feature mode	Trigonometric encoding	Magnitude features	Pooling	Loss	Gaussian noise
Raw 9-axis + padding	raw9_pad	No	No	GMP	Focal Loss	Yes
w/o trigonometric encoding	no_trig	No	Yes	GMP	Focal Loss	Yes
w/o magnitude features	no_mag	Yes	No	GMP	Focal Loss	Yes
w/ GAP	full	Yes	Yes	GAP	Focal Loss	Yes
w/o focal loss	full	Yes	Yes	GMP	Cross Entropy	Yes
w/o Gaussian noise	full	Yes	Yes	GMP	Focal Loss	No
DeM-FCN	full	Yes	Yes	GMP	Focal Loss	Yes

**Table 5:** Ablation study results under LOSO cross-validation.

Variant	Accuracy	Macro precision	Macro recall	Macro F1-score	Macro AUC
Raw 9-axis + padding	0.894 ± 0.121	0.851 ± 0.134	0.832 ± 0.145	0.840 ± 0.138	0.912 ± 0.081
w/o trigonometric encoding	0.955 ± 0.082	0.915 ± 0.115	0.918 ± 0.105	0.911 ± 0.120	0.975 ± 0.042
w/o magnitude features	0.915 ± 0.098	0.868 ± 0.125	0.855 ± 0.130	0.858 ± 0.128	0.935 ± 0.060
w/ GAP	0.921 ± 0.095	0.875 ± 0.112	0.864 ± 0.110	0.868 ± 0.115	0.941 ± 0.055
w/o focal loss	0.935 ± 0.088	0.884 ± 0.105	0.879 ± 0.118	0.880 ± 0.112	0.952 ± 0.048
w/o Gaussian noise	0.948 ± 0.081	0.902 ± 0.094	0.911 ± 0.085	0.905 ± 0.090	0.968 ± 0.035
DeM-FCN	0.966 ± 0.076	0.927 ± 0.203	0.930 ± 0.158	0.916 ± 0.186	0.982 ± 0.050

As shown in Table 5, the raw 9-axis input with zero-padding achieved the weakest performance, with an accuracy of 0.894 ± 0.121, a macro F1-score of 0.840 ± 0.138, and a macro AUC of 0.912 ± 0.081. In contrast, the full DeM-FCN improved these values to 0.966 ± 0.076, 0.916 ± 0.186, and 0.982 ± 0.050, respectively. This clear improvement demonstrates that directly using raw acceleration, gyroscope, and Euler-angle signals is insufficient for optimal subject-independent recognition, while the proposed physics-aware feature representation provides more discriminative and robust kinematic information. The disentangled feature ablation further shows that both trigonometric encoding and magnitude features contribute to the final performance, but their effects are not identical. Removing trigonometric encoding slightly reduced the accuracy to 0.955 ± 0.082, the macro F1-score to 0.911 ± 0.120, and the macro AUC to 0.975 ± 0.042. This suggests that sine and cosine encoding provides additional stability by improving the continuity of angular representation. Since Euler angles may contain boundary discontinuities, the trigonometric transformation maps angular motion into a continuous periodic space that is more suitable for temporal convolution.

Removing magnitude features caused a larger performance drop, with the accuracy decreasing to 0.915 ± 0.098, the macro F1-score decreasing to 0.858 ± 0.128, and the macro AUC decreasing to 0.935 ± 0.060. This indicates that acceleration and gyroscope magnitudes are particularly important for robust subject-independent recognition. These magnitude-based descriptors provide orientation-insensitive motion intensity cues, which are less affected by grip posture, sensor-axis variation, and subject-specific execution style. This is especially relevant for smart dumbbell-based resistance-training recognition, where different users may perform the same exercise with different wrist angles, lifting trajectories, and motion amplitudes.

Overall, the feature-level ablation confirms that trigonometric encoding and magnitude features play complementary roles. Trigonometric encoding mainly improves angular continuity, whereas magnitude features enhance orientation-insensitive motion intensity modeling. The larger degradation observed after removing magnitude features suggests that magnitude-based cues are the more influential component in the current wearable resistance-training setting. Nevertheless, the best performance was achieved only when both feature components were used together, supporting the effectiveness of the complete physics-aware input representation.

#### 3.2.2 Ablation of model components and loss function

The model-level ablations further confirm the contribution of the remaining components in DeM-FCN. Replacing Global Max Pooling with Global Average Pooling reduced the macro F1-score from 0.916 to 0.868, supporting the use of GMP for capturing transient discriminative motion peaks. In resistance-training exercises, discriminative information often appears in short high-response phases, such as peak contraction, direction reversal, and rapid acceleration segments. Compared with GAP, GMP is better suited to preserve these localized peak responses rather than averaging them with less informative frames.

The contribution of the cost-sensitive focal loss was evaluated by replacing it with standard cross-entropy loss while keeping the same feature representation, GMP pooling, and Gaussian noise regularization. As shown in Table 5, the cross-entropy variant achieved an accuracy of 0.935 ± 0.088, a macro F1-score of 0.880 ± 0.112, and a macro AUC of 0.952 ± 0.048. In comparison, the full DeM-FCN with cost-sensitive focal loss achieved a higher accuracy of 0.966 ± 0.076, macro F1-score of 0.916 ± 0.186, and macro AUC of 0.982 ± 0.050. This improvement suggests that the cost-sensitive focal loss contributes to subject-independent recognition by increasing the optimization focus on difficult and low-confidence samples. This is particularly relevant for resistance-training recognition, where some activities have higher biomechanical variability and are more prone to inter-subject confusion. Therefore, the loss function is not merely an auxiliary training choice, but provides measurable performance gains under the LOSO evaluation protocol.

However, the selected class weights should be interpreted as empirically motivated difficulty-aware penalties rather than globally optimized hyperparameters. Although the ablation result supports the usefulness of the proposed loss configuration compared with standard cross-entropy, it does not fully establish the optimality of the specific weight vector. Future work will conduct a more systematic sensitivity analysis of both the class weights and the focusing parameter. Removing Gaussian noise also led to a moderate decrease in performance, with the macro F1-score decreasing from 0.916 to 0.905. This suggests that input-level perturbation provides a useful regularization effect against sensor noise and inter-subject signal variation. The improvement is moderate, but it indicates that noise injection helps the model learn more stable temporal patterns under the LOSO setting.

### 3.3 Model interpretability analysis

To demystify the internal decision-making mechanism of the DeM-FCN and verify that the network learns physically meaningful kinematic patterns rather than spurious background noise, we conducted an interpretability analysis using 1D Class Activation Mapping (1D-CAM).

**Figure 6:**
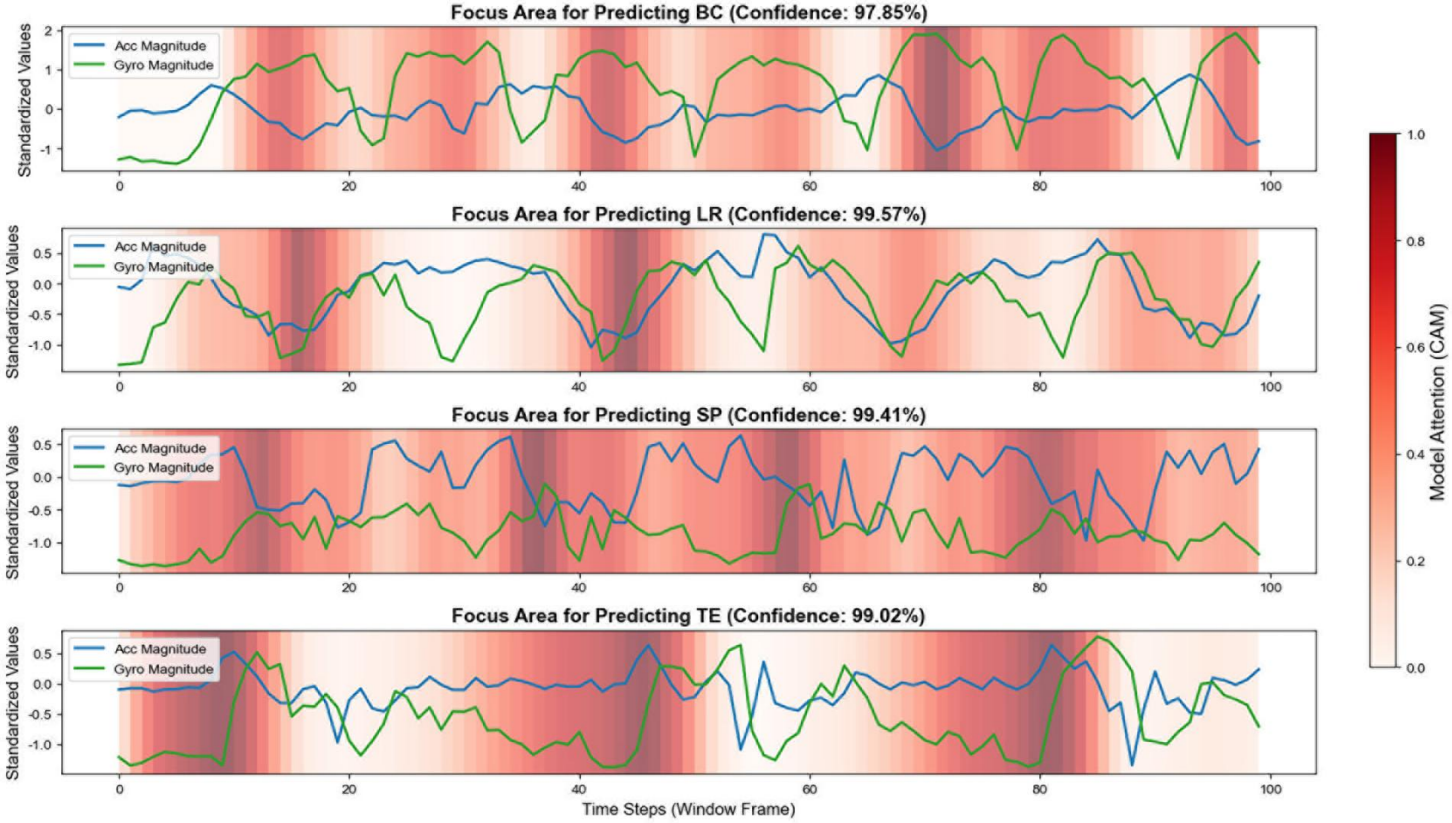
DeM-FCN 1D-CAM interpretability across all actions.

Because the DeM-FCN architecture seamlessly integrates a Global Max Pooling (GMP) layer directly before the final linear classifier, it inherently supports class activation mapping. By projecting the learned weights of the target class from the linear layer back onto the temporal feature maps extracted by the final convolutional block, we generated temporal attention heatmaps. Figure 6 illustrates these heatmaps overlaid on the standardized Euclidean magnitudes of acceleration (blue line) and angular velocity (green line) for four representative samples across a 100-frame sliding window. The color gradient, ranging from white to dark red, quantifies the relative contribution (attention) of each temporal frame to the model's final prediction.

#### 3.3.1 High confidence and dynamic alignment

Across all four actions—Bicep Curl (BC), Lateral Raise (LR), Shoulder Press (SP), and Tricep Extension (TE)—the model exhibits exceedingly high prediction confidence, ranging from 97.85% to 99.57%. More importantly, the 1D-CAM reveals that the network's “visual focus” (indicated by the dark red zones) is not randomly distributed, nor does it fixate on the relatively flat, static resting phases. Instead, the model's attention is strongly, temporally correlated with the critical dynamic transitions of the exercises.

#### 3.3.2 Biomechanical feature isolation

A closer inspection of the specific exercises reveals profound alignment with human biomechanics:

1. Lateral Raise (LR) and Bicep Curl (BC): In the subplots for LR and BC, the deepest red attention peaks align precisely with the local minima or sharp inflection points of the Gyro Magnitude (green curve). From a biomechanical perspective, these moments correspond to the apex of the concentric contraction—the point where the limb reaches its maximum range of motion (e.g., arms fully raised or elbow fully flexed) and momentarily decelerates to zero before initiating the eccentric lowering phase. The model correctly identifies these zero-crossing angular velocity transitions as the most discriminative signatures for these exercises.
2. Tricep Extension (TE) and Shoulder Press (SP): For TE and SP, the model heavily weighs the regions characterized by explosive, high-amplitude fluctuations in both acceleration and angular velocity. In the TE subplot, the dark red bands tightly wrap around the massive spikes in sensor values, successfully capturing the explosive extension of the elbow joint under load.

#### 3.3.3 Validation of the Gmp layer's translation invariance

The distribution of the dark red zones across different parts of the 100-frame windows serves as empirical proof of the Global Max Pooling (GMP) layer's temporal translation invariance. The exact start and end points of an exercise cycle vary across windows due to the 50% overlap sliding step. However, the 1D-CAM demonstrates that the DeM-FCN acts as an intelligent “peak-detector.” As long as the salient kinematic signatures (the dark red phases) occur anywhere within the window, the model successfully locks onto them and makes a highly confident prediction. This interpretability analysis substantiates that the DeM-FCN does not merely memorize localized data points but genuinely comprehends the underlying spatio-temporal physics of human resistance training.

### 3.4 Computational cost and edge deployment efficiency analysis

To provide an indicative assessment of computational feasibility, we profiled the proposed DeM-FCN on a general-purpose workstation equipped with an AMD Ryzen 7 CPU and 32 GB RAM, together with an NVIDIA RTX 3060 GPU for throughput reference. These experiments were intended to estimate computational complexity, memory footprint, and inference latency under controlled hardware conditions. Therefore, the results should be interpreted as indicative computational profiling rather than complete embedded-device validation. Actual performance on microcontrollers, smartwatches, or wearable SoCs may differ because of hardware-specific memory hierarchy, numerical precision, runtime libraries, power-management policies, and sensor I/O overhead. The comprehensive profiling results are detailed in Table 6.

1. Ultra-Lightweight Complexity (FLOPs and Parameters): The primary bottleneck for deploying deep learning models on wearable sensors is the strict limitation on computational power and battery life. The proposed DeM-FCN mitigates this by functioning as a highly specialized 1D temporal feature extractor. With a total parameter count bounded at 73.7 K, the architecture requires only ∼14.84 Million Floating-Point Operations (FLOPs) to process a standard sliding window (100 frames × 14 channels). Compared with more complex Transformer-based or heavy 2D convolutional architectures, this FLOPs profile suggests a lower computational burden and potentially favorable suitability for continuous monitoring. However, actual energy efficiency still needs to be verified on the specific embedded wearable hardware.
2. Minimal Memory Footprint: For embedded hardware and typical edge nodes, non-volatile storage and SRAM are extremely scarce. The compiled DeM-FCN model requires only ∼0.29 MB (290 KB) of non-volatile storage in its standard 32-bit floating-point (FP32) format. Furthermore, the peak RAM allocation during a single forward-pass inference remains strictly under 2.0 MB. This compact memory footprint suggests potential feasibility for deployment on wearable nodes and may facilitate future firmware-level integration. Nevertheless, actual deployment feasibility depends on device-specific memory constraints, runtime support, numerical precision, and communication overhead.
3. Real-Time Inference and Throughput: Real-time, continuous activity monitoring demands low-latency execution to provide users with immediate feedback. During baseline profiling on the tested CPU platform, the inference latency was measured at less than 2.5 ms per window, corresponding to a throughput of more than 400 windows per second under this hardware setting. Given a 50-frame sliding step, this result suggests that DeM-FCN has sufficient computational headroom for real-time processing in the tested environment. However, this latency should not be interpreted as direct evidence of zero-lag execution on wearable edge hardware. End-to-end latency on embedded devices may be affected by sensor I/O, memory transfer, runtime optimization, quantization strategy, and power-management policies. Future work will deploy DeM-FCN on representative wearable or microcontroller platforms to evaluate practical latency, memory behavior, energy consumption, and long-term operating stability.

**Table 6:** Computational complexity and edge deployment efficiency benchmarks.

Metric category	Specific indicator	Value/estimation
Model complexity	Total Parameters	73,732 (∼73.7 K)
	FLOPs (per 100-frame window)	∼14.84 M
	MACs (per 100-frame window)	∼7.42 M
Edge storage & memory	Model Weights Size (FP32)	∼0.29 MB
	Peak RAM Usage (Single Inference)	< 2.0 MB
Execution efficiency	Inference Latency (Single Window)	< 2.5 ms
	Processing Throughput	> 400 windows/sec

### 3.5 Comparative analysis of models

To evaluate the proposed DeM-FCN framework, we benchmarked it against five recent advanced edge HAR models. The comparison encompasses both classification performance and computational efficiency (Model Size and CPU Inference Latency), as detailed in Table 7.

1. Accuracy vs. Complexity Trade-off: DeM-FCN delivers highly competitive performance, ranking in the top three across all classification metrics. While AlMuhaideb et al. achieves a marginally higher AUC (0.985 vs. our 0.982), they rely on a heavyweight CNN + LSTM architecture. This recurrent design incurs a severe computational penalty (2.10 MB size, 45.0 ms latency). Our pure 1D-FCN architecture entirely avoids recurrent overhead, securing near-identical predictive power with a fraction of the computational cost.
2. Superior Edge Deployability: Conversely, methods explicitly targeting ultra-low-power TinyML (Liu et al., Malche, Trotta et al.) achieve highly compressed memory profiles (< 0.2 MB) through extreme quantization or traditional machine learning (e.g., SOMs). However, these strict constraints force compromises: they either suffer significant drops in accuracy (e.g., 0.935 for Trotta et al.) or struggle with sluggish sequential processing delays (e.g., 85.0 ms inference for Malche).
3. Favorable Accuracy-Efficiency Trade-off: The proposed DeM-FCN provides a favorable balance between recognition performance and computational cost under the current experimental setting. Compared with the most accurate baseline listed in Table 7, the model achieved comparable predictive performance with a smaller model size and lower CPU latency in the reported profiling results. Note that all baseline results in Table 7 are cited from original publications rather than re-trained on our dataset, owing to inconsistent experimental setups across studies. However, the baseline results may differ in datasets, implementation details, hardware platforms, and evaluation protocols. Therefore, DeM-FCN is positioned as an efficient and edge-oriented HAR backbone. Further standardized benchmarking and embedded-device validation are needed to establish its deployment advantages more rigorously. A recent lightweight HAR framework, PWMF-ResMiniNet (23), demonstrates impressive computational efficiency for multi-sensor general activity recognition. While it achieves 30.4 K parameters and 3.77 M FLOPs per modality, four key differences justify the unique advantages of DeM-FCN for single-sensor resistance-training recognition: First, sensor configuration requirements differ fundamentally. ResMiniNet's peak performance relies on three synchronized sensors placed at the chest, wrist, and ankle. In contrast, DeM-FCN operates exclusively on a single wrist-mounted 9-axis IMU integrated into the dumbbell handle, which is the only commercially viable configuration for low-cost, user-friendly smart fitness equipment. Second, end-to-end latency considerations are critical for real-time applications. The reported 3.77 M FLOPs only include 2D CNN inference and exclude the computationally expensive Gramian Angular Field (GAF) time-series imaging preprocessing step, which adds approximately 8 M additional FLOPs per window. DeM-FCN processes raw 1D inertial signals directly without intermediate transformations, resulting in lower actual end-to-end inference latency. Third, architectural optimizations are task-specific. DeM-FCN's Global Max Pooling layer is explicitly designed to capture the transient force peaks and rapid joint angle changes that define resistance-training movements. This design delivers a higher macro AUC (0.982) on our target task compared to the 0.967 AUC reported by ResMiniNet on the general-purpose PAMAP2 dataset. Finally, deployment simplicity and robustness are prioritized. DeM-FCN adopts a pure 1D convolutional architecture with no residual connections, multi-branch fusion modules, or sequential dependencies. This design simplifies firmware implementation and improves numerical stability on embedded hardware with limited floating-point precision. These differences demonstrate that optimal accuracy-efficiency trade-offs are inherently task-dependent. While ResMiniNet excels at multi-sensor general daily activity recognition, DeM-FCN provides a more practical and effective solution for single-sensor wearable resistance-training applications.

**Table 7:** Performance and efficiency comparison with recent edge HAR models.

Method	Backbone architecture	Overall accuracy	Macro F1-score	Macro AUC	Model size (MB)	Inference latency (ms)
Duarte et al.	Edge CNN (Off-the-person)	0.951	0.898	0.965	0.45	18.5
Liu et al.	Quantized Edge CNN	0.948	0.905	0.961	0.18	25
Malche	TinyML NN	0.952	0.93	0.955	0.06	85
Trotta et al.	Feat. Selection + SOM	0.935	0.88	0.94	0.12	12
AlMuhaideb et al.	CNN + LSTM Variant	0.971	0.942	0.985	2.1	45
DeM-FCN (Ours)	1D-FCN + GMP	0.966	0.916	0.982	0.29	2.5

### 3.6 Performance evaluation on public datasets

To validate the generalized feature extraction capabilities of the proposed DeM-FCN architecture, we conducted subject-independent evaluations on two established benchmark datasets: PAMAP2 (Physical Activity Monitoring Dataset 2) and MHEALTH (Mobile HEALTH dataset). These datasets encompass a wide range of daily living activities (ADLs) and sports, presenting variations in sensor placement, sampling rates, and exercise types compared to the proprietary dataset.

#### 3.6.1 Validation of the Gmp layer's translation invariance

Given the differences in hardware specifications across the datasets, a Spatio-Temporal Alignment Module was implemented to ensure the input data format remained compatible with the DeM-FCN backbone without modifying its internal convolutional parameters.

1. Adaptation for PAMAP2: The PAMAP2 dataset provides synchronized IMU data at a 100-Hz sampling rate. However, according to the dataset documentation, the orientation (Euler angle) data is invalid. To maintain the 14-channel input structure, Modality Padding was employed, where the missing orientation channels were zero-padded. The model utilized the triaxial acceleration and gyroscope data from the dominant wrist IMU to compute the Euclidean norms and extract kinematic features.
2. Adaptation for MHEALTH: The MHEALTH dataset records motion signals at a sampling rate of 50 Hz. To align this with the DeM-FCN's temporal receptive field, Temporal Rescaling was implemented by adjusting the sliding window to 50 frames (equivalent to 1 second). Acceleration units were converted to g-units, and zero-padding was applied to the absent Euler angle channels. This configuration requires the model to isolate features primarily from raw inertial sequences and their calculated magnitudes.

The Leave-One-Subject-Out (LOSO) cross-validation protocol was executed independently on both datasets to assess subject-independent generalization.

#### 3.6.2 Experimental results and kinematic mechanism analysis

Table 8 shows that DeM-FCN achieved a macro AUC of 0.972 ± 0.051 across the 12 evaluated activities on the PAMAP2 dataset. The hard classification metrics were lower than those obtained on the custom smart dumbbell dataset, with a macro F1-score of 0.787 ± 0.218. The high macro AUC suggests that the model retained informative class-ranking ability in a more complex activity setting. Dynamic activities such as walking, cycling, Nordic walking, stair ascent, stair descent, vacuum cleaning, and ironing showed stable performance. This stability indicates that the Global Max Pooling layer effectively extracts translation invariant features, identifying kinematic transitions independent of their exact temporal position within the sliding window. Static or quasi-static postures, along with high impact exercises like rope jumping, exhibited lower F1-scores and larger standard deviations. The results for static postures highlight the operational limitations caused by the absence of 3D spatial reference frames. Since valid Euler angle cues are missing and replaced by zero padding, the model relies primarily on the gravity component distribution across the accelerometer axes. Baseline sensor noise and exact limb angles vary widely among unseen subjects in the LOSO-CV protocol. Consequently, the lack of explicit orientation information makes it difficult for the model to establish stable hard classification thresholds for activities that differ mainly in body orientation. The high standard deviation for rope jumping suggests that subject specific execution styles and sample distribution shifts strongly affect classification stability. The discrepancy between macro F1-score and AUC provides additional context for interpreting the public dataset results. The high AUC values for lying (0.982) and sitting (0.965) suggest that the model retained useful ranking ability for these classes, even though the corresponding hard-label metrics were less stable. However, this should not be interpreted as definitive evidence of a fully separable feature space. In the absence of explicit calibration analysis, the lower F1-scores may be associated with class overlap, subject-specific distribution shifts, threshold sensitivity, and missing orientation cues. These results suggest that DeM-FCN can be applied to public IMU-based HAR data, while its hard prediction performance remains affected when posture-related orientation information is unavailable.

**Table 8:** Subject-independent evaluation results on the PAMAP2 dataset.

Category	Accuracy	Precision	Recall	F1-Score	AUC
Overall/macro average	0.823 ± 0.180	0.809 ± 0.199	0.808 ± 0.186	0.787 ± 0.218	0.972 ± 0.051
Lying	0.938 ± 0.041	0.644 ± 0.306	0.686 ± 0.398	0.623 ± 0.363	0.982 ± 0.022
Sitting	0.913 ± 0.048	0.601 ± 0.242	0.736 ± 0.220	0.623 ± 0.196	0.965 ± 0.029
Standing	0.963 ± 0.027	0.892 ± 0.112	0.725 ± 0.282	0.755 ± 0.222	0.961 ± 0.053
Walking	0.976 ± 0.038	0.836 ± 0.329	0.827 ± 0.318	0.831 ± 0.322	0.996 ± 0.007
Running	0.989 ± 0.021	0.824 ± 0.369	0.829 ± 0.371	0.826 ± 0.370	0.887 ± 0.252
Cycling	0.969 ± 0.043	0.783 ± 0.349	0.778 ± 0.320	0.757 ± 0.313	0.996 ± 0.005
Nordic walking	0.983 ± 0.038	0.853 ± 0.348	0.850 ± 0.347	0.852 ± 0.348	0.979 ± 0.046
Ascending stairs	0.986 ± 0.010	0.911 ± 0.086	0.842 ± 0.271	0.832 ± 0.221	0.978 ± 0.050
Descending stairs	0.958 ± 0.067	0.855 ± 0.277	0.775 ± 0.195	0.755 ± 0.235	0.990 ± 0.011
Vacuum cleaning	0.967 ± 0.041	0.799 ± 0.210	0.893 ± 0.209	0.839 ± 0.200	0.972 ± 0.068
Ironing	0.960 ± 0.036	0.752 ± 0.290	0.831 ± 0.317	0.788 ± 0.300	0.969 ± 0.070
Rope jumping	0.991 ± 0.011	0.649 ± 0.459	0.619 ± 0.449	0.631 ± 0.451	0.927 ± 0.126

As shown in Table 9, DeM-FCN achieved an overall accuracy of 0.768 ± 0.109 and a macro F1-score of 0.741 ± 0.109 on the MHEALTH dataset. This lower macro F1-score reflects the increased complexity introduced by broader activity categories, sensor domain differences, and the missing Euler angle channels. Despite this, the macro AUC of 0.981 ± 0.020 suggests that the model continued to produce informative class-ranking scores across activity classes. Activities characterized by clear dynamic temporal patterns achieved strong recognition performance. Examples include the frontal elevation of arms, cycling, and jump front and back. In contrast, activities such as sitting and relaxing, lying down, waist bends forward, and knees bending proved more difficult to distinguish. These specific activities either contain weak inertial fluctuations or share partial motion trajectories with other classes. The results suggest that DeM-FCN is more suitable for activities with distinct temporal motion signatures. Static postures and short transient movements remain more sensitive to sensor placement variations, subject-specific execution differences, and possible decision-threshold instability.

**Table 9:** Subject-independent evaluation results on the MHEALTH dataset.

Category	Accuracy	Precision	Recall	F1-Score	AUC
Overall/macro average	0.768 ± 0.109	0.760 ± 0.107	0.778 ± 0.105	0.741 ± 0.109	0.981 ± 0.020
Standing still	0.981 ± 0.034	0.833 ± 0.312	0.900 ± 0.300	0.858 ± 0.301	0.990 ± 0.029
Sitting and relaxing	0.946 ± 0.043	0.498 ± 0.445	0.600 ± 0.490	0.533 ± 0.451	0.973 ± 0.041
Lying down	0.948 ± 0.043	0.600 ± 0.436	0.623 ± 0.466	0.571 ± 0.421	0.979 ± 0.042
Walking	0.968 ± 0.056	0.881 ± 0.262	0.802 ± 0.283	0.827 ± 0.264	0.984 ± 0.041
Climbing stairs	0.944 ± 0.078	0.721 ± 0.283	0.898 ± 0.245	0.788 ± 0.264	0.962 ± 0.100
Waist bends forward	0.942 ± 0.025	0.679 ± 0.288	0.514 ± 0.367	0.517 ± 0.308	0.978 ± 0.021
Frontal elevation of arms	0.983 ± 0.032	0.904 ± 0.193	0.994 ± 0.011	0.934 ± 0.128	1.000 ± 0.001
Knees bending (crouching)	0.921 ± 0.039	0.517 ± 0.253	0.654 ± 0.359	0.538 ± 0.274	0.956 ± 0.042
Cycling	0.990 ± 0.029	0.898 ± 0.299	0.900 ± 0.300	0.899 ± 0.300	1.000 ± 0.000
Jogging	0.956 ± 0.059	0.799 ± 0.308	0.718 ± 0.421	0.700 ± 0.386	0.963 ± 0.077
Running	0.957 ± 0.058	0.792 ± 0.319	0.812 ± 0.337	0.773 ± 0.310	0.986 ± 0.032
Jump front & back	0.998 ± 0.004	1.000 ± 0.000	0.926 ± 0.136	0.956 ± 0.082	1.000 ± 0.000

## 4 Discussion

The proposed DeM-FCN achieved strong subject-independent recognition on the custom smart dumbbell dataset, with an accuracy of 0.966, macro F1-score of 0.916, and macro AUC of 0.982 under the LOSO protocol. Since each test subject was excluded from training, these results indicate that the model learned transferable motion patterns rather than subject-specific features. Meanwhile, the model remained lightweight, with only 73.7 K parameters, 14.84 M FLOPs, and a model size of 0.29 MB, showing a favorable accuracy-efficiency trade-off for wearable resistance-training recognition. The aggregated confusion matrix further revealed that the remaining errors were not random. Bicep Curl, Shoulder Press, and Tricep Extension showed high separability, whereas Lateral Raise was the main source of confusion. This is biomechanically reasonable because Lateral Raise is more affected by shoulder abduction amplitude, elbow flexion, torso compensation, wrist posture, and movement momentum. Therefore, the perfect scores observed in several subjects should not be interpreted as evidence of dataset triviality. Instead, they reflect separable kinematic patterns in part of the cohort, while the Lateral Raise-related errors indicate that inter-subject variability remains a practical challenge.

The refined ablation study confirmed the effectiveness of the proposed feature representation. Compared with the raw 9-axis input, the full DeM-FCN improved accuracy from 0.894 to 0.966 and macro F1-score from 0.840 to 0.916. The disentangled feature ablation showed that trigonometric encoding and magnitude features provide complementary benefits. Removing trigonometric encoding caused only a slight decrease, suggesting that sine/cosine encoding improves angular continuity. Removing magnitude features caused a larger drop, indicating that acceleration and gyroscope magnitudes are more important for capturing orientation-insensitive motion intensity and reducing sensitivity to grip posture and sensor-axis variation. The model-level ablation also supported the main architectural choices. Replacing GMP with GAP reduced macro F1-score from 0.916 to 0.868, confirming the advantage of GMP in capturing transient discriminative peaks during resistance-training movements. Replacing the cost-sensitive focal loss with cross-entropy reduced accuracy from 0.966 to 0.935 and macro F1-score from 0.916 to 0.880, suggesting that difficulty-aware optimization improves hard-class recognition. However, the class weights remain empirically specified, and future work should further examine weight sensitivity and adaptive reweighting strategies. Removing Gaussian noise also slightly reduced performance, indicating a moderate regularization effect. This interpretation is also consistent with recent biomechanics-informed interpretable machine learning research. For example, Mănescu et al. (24) used simulation-derived biomechanical features for hamstrings-quadriceps imbalance detection in running and showed that physically meaningful biomechanical descriptors can support transparent and interpretable decision-making in sports-related movement analysis. In this context, the trigonometric encoding and magnitude features used in DeM-FCN are not only additional signal channels, but also interpretable descriptors of angular continuity and motion intensity.

Public dataset experiments provided a broader assessment of generalization. On PAMAP2 and MHEALTH, DeM-FCN achieved macro AUC values of 0.972 and 0.981, respectively, but the macro F1-scores decreased to 0.787 and 0.741. This discrepancy suggests that the convolutional backbone retained useful class-ranking ability, while hard-label decisions remained more sensitive to class overlap, threshold selection, subject-specific distribution shifts, and missing orientation information. Therefore, the high AUC values should not be overinterpreted as evidence of well-calibrated probabilities or fully separable class distributions. Broad daily-activity recognition remains more challenging because of greater activity diversity, different sensor placements, sampling-rate differences, and incomplete modality information.

Several limitations should be acknowledged. First, the custom dataset includes only 15 subjects and four resistance-training exercises. Although LOSO validation provides a strict subject-independent evaluation, the dataset size and activity scope still constrain the strength of the conclusions. Second, the computational evaluation was performed on a general-purpose CPU/GPU platform rather than on actual wearable edge hardware. Therefore, the reported latency and memory results should be interpreted as indicative profiling rather than complete embedded-device validation. Third, the window size and overlap ratio were fixed according to practical considerations of temporal coverage, boundary robustness, sample efficiency, and inference frequency, but systematic sensitivity analysis was not conducted. Fourth, the class weights in the cost-sensitive focal loss were empirically specified, and explicit probability calibration analysis was not performed. Future work will focus on larger-scale data collection, real-device deployment, window-parameter sensitivity analysis, adaptive loss weighting, calibration-aware evaluation, sensor-placement robustness, and lightweight subject-specific calibration. Fifth, the cross-subject “classification paradox” remains a challenge (e.g., perfect AUC of 1.0 for subjects YWH and TP) but exhibits suboptimal hard classification accuracy (e.g., F1-score below 0.7 for the same subjects), as clearly observed in our LOSO validation results. The cause is that the model effectively learns relative differences between activities (enabling strong ranking ability), but a single global decision threshold cannot adapt to the significant inter-subject variations in movement amplitude, speed, and exertion patterns. This is a fundamental challenge shared by all subject-independent HAR studies. Future work will investigate lightweight personalized calibration techniques that adjust decision thresholds based on 1-2 standard movements from new users, without requiring full model retraining.

## 5 Conclusion

This study proposed DeM-FCN, a lightweight purely convolutional framework for wearable resistance-training activity recognition using smart dumbbell inertial signals. By integrating physics-aware feature engineering, Global Max Pooling, Gaussian noise regularization, and cost-sensitive focal loss, the model achieved strong subject-independent performance under LOSO cross-validation, with an accuracy of 0.966, macro F1-score of 0.916, and macro AUC of 0.982, while requiring only 73.7 K parameters, 14.84 M FLOPs, and 0.29 MB storage. The refined ablation study confirmed that trigonometric encoding and magnitude features provide complementary benefits. Magnitude features were especially important for capturing orientation-insensitive motion intensity, while GMP and focal loss further improved transient feature extraction and hard-class recognition. Public evaluations on PAMAP2 and MHEALTH suggested that DeM-FCN retains useful class-ranking ability on broader IMU-based HAR tasks, although the reduced macro F1-scores indicate that hard-label daily activity recognition remains challenging due to activity diversity, sensor differences, and missing modality information. Overall, DeM-FCN provides a favorable accuracy-efficiency trade-off for edge-oriented wearable resistance-training recognition under the current experimental setting. However, the conclusions remain constrained by dataset size, activity scope, fixed window parameters, and the absence of real embedded-device validation. Future work will focus on larger datasets, real-device deployment, window-parameter sensitivity analysis, adaptive loss weighting, calibration-aware evaluation, and improved robustness to sensor-placement variability.

## Data Availability

The datasets presented in this study can be found in online repositories. The names of the repository/repositories and accession number(s) can be found below: Data available at Zenodo: https://doi.org/10.5281/zenodo.19501489.
